# Characterizing Long Transients in Consumer–Resource Systems With Group Defense and Discrete Reproductive Pulses

**DOI:** 10.1007/s11538-022-01059-7

**Published:** 2022-08-14

**Authors:** Jorge Arroyo-Esquivel, Alan Hastings, Marissa L. Baskett

**Affiliations:** 1 Department of Mathematics, University of California, Davis, CA 95616 USA; 2 Department of Environmental Science and Policy, University of California, Davis, CA 95616 USA

**Keywords:** Long transients, Consumer–resource

## Abstract

During recent years, the study of long transients has been expanded in ecological theory to account for shifts in long-term behavior of ecological systems. These long transients may lead to regime shifts between alternative states that resemble the dynamics of alternative stable states for a prolonged period of time. One dynamic that potentially leads to long transients is the group defense of a resource in a consumer–resource interaction. Furthermore, time lags in the population caused by discrete reproductive pulses have the potential to produce long transients, either independently or in conjunction to the transients caused by the group defense. In this work, we analyze the potential for long transients in a model for a consumer–resource system in which the resource exhibits group defense and reproduces in discrete reproductive pulses. This system exhibits crawl-by transients near the extinction and carrying capacity states of resource, and a transcritical bifurcation, under which a ghost limit cycle appears. We estimate the transient time of our system from these transients using perturbation theory. This work advances an understanding of how systems shift between alternate states and their duration of staying in a given regime and what ecological dynamics may lead to long transients.

## Introduction

One of the goals of mathematical modeling of ecological systems is to understand the fate or long-term dynamics of such system. The main method to study such fate has been through the analysis of the attractors in a model (Ives and Carpenter 2007). Recent years have seen an increase in the interest of understanding non-attractor dynamics (hereafter transients) of the models, especially those that resemble an attractor for a long period of time (hereafter long transients) (Hastings et al. 2018). Long transients have gained recognition as a theoretical tool to better describe population dynamics by allowing the study of dynamics that occur in a more biologically relevant timeframe (Morozov et al. 2020). In addition, an understanding of long transients can inform conservation and natural resource management goals. For example, identifying that a positively valued long-term behavior observed in nature is actually a long transient and what causes it can guide management to prolong it (Francis et al. 2021).

Long transients often appear in the presence of a “small” (close to zero) parameter in the model (Morozov et al. 2020). One of the main challenges of identifying long transients is identifying such a small parameter, which may be a function of the biologically reasonable parameters, and thus may not be easily interpretable. For example, in ghost attractors, this small parameter is the difference between a bifurcation parameter and its bifurcation value (Morozov et al. 2020). While varying the parameter past such bifurcation leads to the destruction of an attractor, small differences the transient dynamics will resemble the attractor. In crawl-by attractors, the small parameter is determined by the degree to which the trajectory of the system is parallel to the stable manifold of a saddle node equilibrium at a given time (Morozov et al. 2020). In this case the system will behave similarly to such a stable manifold for a prolonged period of time before the unstable part of the trajectory leads to a change in the system behavior.

One behavior that has been demonstrated to lead to long transients in consumer–resource systems is group defense (Venturino and Petrovskii 2013). Group defense is a behavior where a resource population reduces the risk of individuals being predated by protecting each other. This behavior occurs in diverse animal taxa, which produce early-warning signals to detect predators, as is the case of colonial spiders (Uetz et al. 2002), birds, (Robinson 1985), and mammals (Ebensperger and Wallem 2002). This behavior also occurs in producers such as kelp, where high densities of kelp lead to an increase in predators of kelp grazers, which induces cryptic behavior on such grazers and thus reduces grazing intensity (Karatayev et al. 2021).

Group defense transients might also depend on lags in population growth caused by discrete reproductive pulses. In some taxa that exhibit group defense, adult stages of the population may reproduce in discrete, seasonal pulses, such as is the case of kelp (Karatayev et al. 2021) or bees (Kastberger et al. 2008). This can provide individuals to a population decades after stressful events which cause population declines, such as competitive exclusion of pioneer species in tropical rain forests (Dalling and Brown 2009), or extreme weather events in phytoplankton (Ellegaard and Ribeiro 2018).

In this paper we characterize the long transients in a consumer–resource with both group defense and reproductive pulses. We first construct the model that describes a consumer–resource interaction where the resource exhibits group defense and has discrete reproductive pulses. Then, to illustrate the long transients present in this model, we identify a small parameter that describes each of the transients (crawl-by and ghost attractor), and we use this parameter to calculate the time the system remains in this long transient (hereafter transient time). Finding approximations for these parameters and transient times provides biological insight into how these long transients may arise in natural systems with the modeled dynamics. We conclude this paper with a discussion of these results and their biological implications.

## Model

In this section we construct a consumer–resource model with group defense and discrete reproductive pulses. We previously explored a spatial, non-smooth version of this model to understand spread of kelp being grazed by urchins (Arroyo-Esquivel et al. 2021). We consider the dynamics of adult consumer *P* and adult resource *N* densities through time. Adults of population $i=P,N$ experience a natural mortality at a rate $d_i$. In addition, consider that consumers consume resource following a unimodal Type IV Holling functional response that represents group defense with a decline in consumption at high resource densities (Andrews 1968). We let $\gamma _N$ be the attack rate of the consumer, and the maximum per-capita resource consumption occurs when $N=\frac{1}{\sqrt{\sigma _N}}$.

Reproduction and recruitment of juvenile stages occur at discrete points in time. We model this recruitment as an impulsive differential equation. Let $t=m$ be the periods at which the offspring recruit to the population. The number of consumer recruits is proportional to the amount of resource consumed at time $t=m$ with proportionality constant $\gamma _P$. Resource produce a per-capita number *R* of recruits. We assume that $R>1-\exp (-d_N)$ in order to have a self-replenishing resource in the absence of consumers. For predation, a fraction of those offspring survive consumption with a probability following an exponential distribution with mean $\frac{1}{\gamma _S}$. Resource offspring also survive intracompetition from adults with carrying capacity proportional to $\frac{1}{\beta }$.

Then, given $P_{m+1}^-$ as the density of consumers before the pulse and $P_{m+1}^+$ its density after the pulse (with analogous notation for resource, $N_{m+1}^-$ and $N_{m+1}^+$), the dynamics of the adult consumer and resource populations satisfy the following system of impulsive differential equations:

1 $$\begin{aligned} \begin{aligned} \frac{\mathrm{d} P}{\mathrm{d} t}&=-d_PP,\\ \frac{\mathrm{d} N}{\mathrm{d} t}&=-\frac{\gamma _N PN}{1+\sigma _N N^2}-d_NN,\\ P^+_{m+1}&=P^-_{m+1}+\gamma _P\frac{P^-_{m+1}N^-_{m+1}}{1+\sigma _N N^{-2}_{m+1}},\\ N^+_{m+1}&=N^-_{m+1}+R\frac{\exp \left( -\gamma _S P^-_{m+1}\right) }{1+\beta N^-_{m+1}}N^-_{m+1}. \end{aligned} \end{aligned}$$

We next transform Model 1 into a discrete-time model. We can rewrite the continuous part of the Model 1 as

2 $$\begin{aligned} \begin{aligned} \frac{1}{P}\frac{\mathrm{d} P}{\mathrm{d} t}&=-d_P,\\ \frac{1}{N}\frac{\mathrm{d} N}{\mathrm{d} t}&=-\frac{\gamma _N P}{1+\sigma _N N^2}-d_N. \end{aligned} \end{aligned}$$

Following the derivation of Cui et al. (2016), we discretize System 2 as

3 $$\begin{aligned} \begin{aligned} P_{m+1}&=P_m\exp (-d_P),\\ N_{m+1}&=N_m\exp (-d_N)\exp \left( -\frac{\gamma _N\exp (-d_P) P_m}{1+\sigma _N N_m^2}\right) . \end{aligned} \end{aligned}$$

By taking $\delta _P=\exp (-d_P),\delta _N=\exp (-d_N)$ and using $P_{m+1}^-=P_m$ and $N_{m+1}^-=N_m$ as described in System 1, we arrive the following discrete-time model:

4 $$\begin{aligned} \begin{aligned} P_{m+1}&=\delta _P P_m+\gamma _P\frac{P_{m}N_m}{1+\sigma _N N_{m}^2},\\ N_{m+1}&=\delta _NN_m\exp \left( -\frac{\gamma _N\delta _P P_m}{1+\sigma _N N_m^2}\right) \\ {}&\quad +R\frac{\exp \left( -\gamma _S P_m\right) }{1+\beta N_m}N_m. \end{aligned} \end{aligned}$$

To simplify our analysis, we will study a nondimensional version of the model. For each *m*, let $p_m=\gamma _SP_m,n_m=\beta N_m$. Then, if $\gamma _p=\gamma _P/\beta ,\gamma _n=\gamma _N\delta _P/\gamma _S,\sigma =\sigma _N/\beta ^2$, our nondimensional version of the model is

5 $$\begin{aligned} \begin{aligned} p_{m+1}&=\delta _p p_m+\gamma _p\frac{p_{m}n_m}{1+\sigma n_{m}^2},\\ n_{m+1}&=\delta _nn_m\exp \left( -\frac{\gamma _n p_m}{1+\sigma n_m^2}\right) +Rn_m\frac{\exp \left( - p_m\right) }{1+n_m}. \end{aligned} \end{aligned}$$

Note that we have also changed the indices of $\delta _i$ and $k_i$ in order to preserve clarity.

## Analysis and Results

In this section we characterize the dynamics of Model 5 and its potential for long transient dynamics. We identify two different classes of long transients, a crawl-by transient around the extinction of resource and another around the carrying capacity of the resource, and a ghost consumer–resource cycle. To illustrate the transients identified and test the accuracy of our analytical approximations, we also characterize all transients numerically by iterating the logarithm of Model 5 in Julia, where the used, fixed parameters and initial conditions are specified as relevant to each analysis below. Based on preliminary numerical simulations in double-precision floating-point numbers, we find that iterating the logarithm of Model 5 instead of the original model prevents numerical instabilities potentially occurring at long-transients near zero by reducing the range of the derivative near zero. The source code for these simulations can be found in https://github.com/jarroyoe/characterizing-transients. We analytically derive approximations for the transient time of the crawl-by transients using perturbation theory, while we numerically analyze the ghost attractor transient time by regressing the transient time using a power law, which we describe further in Section 3.b.

Before we characterize these long transients, we first analyze the equilibria of the model. This model has up to four biologically relevant fixed points: a resource-only carrying capacity equilibrium $(0,n^*)$, an unstable extinction equilibrium (0, 0), and two possible unstable coexistence saddle equilibria $(p^{\vee \wedge },n^{\vee \wedge })$. We assume that the carrying capacity of resource is greater than the density at which consumption growth is its highest, i.e., $n^*>1/\sqrt{\sigma }$, such that group defense is relevant to resource populations below carrying capacity. Under this condition, the equilibria $(0,n^*)$ and $(p^\wedge ,n^\wedge )$ go through a transcritical bifurcation at

6 $$\begin{aligned} \gamma _p^*=(1-\delta _p)\frac{1+\sigma n^{*2}}{n^*}. \end{aligned}$$

In this case, the equilibria $(0,n^*)$ is stable for $\gamma _p<\gamma _p^*$ and unstable for $\gamma _p>\gamma _p^*$, and the equilibrium $(p^{\wedge },n^{\wedge })$ is unstable for $\gamma _p<\gamma _p^*$ and is not in the first quadrant (i.e., $\mathbb {R}^2_+$) for $\gamma _p>\gamma _p^*$. See Appendix A for the expressions of these equilibria and their stability. This analysis allows us to better understand the nature of the transients we have identified.

### Crawl-by Transients

**Fig. 1:**
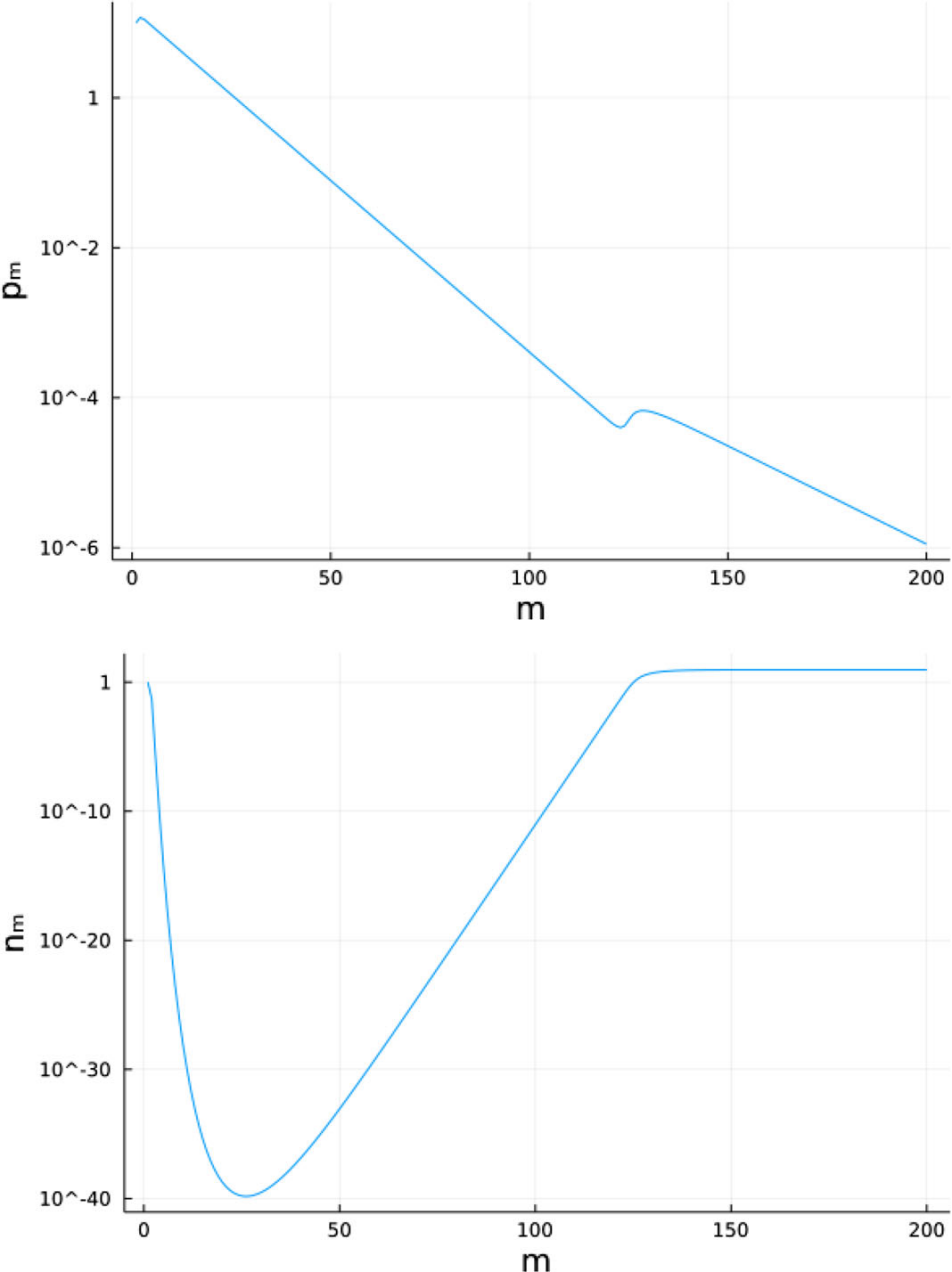
Time series in logarithmic scale of **a** consumers $p_m$ and **b** resource $n_m$ following System 5 for 200 time steps (*m*). In this figure, $p_0=10,n_0=1,\delta _p=0.9,\gamma _p=1,\sigma =2.67,\delta _n=0.8,\gamma _n=1,R=2$.

Although the coextinction equilibrium is a saddle in the *n*-direction (which implies that *n* will stay above 0), System 5 can resemble a system where the resource is extinct for a long period of time when consumer density is high (Fig. 1). This is an example of a long crawl-by transient. We determine how prevalent this behavior is in the following theorem, proven in Appendix B.

#### Theorem 1

Let $\varepsilon \ll 1$. If $p_0$ is of order $\varepsilon ^{-1}$ and $n_0$ of order 1, then System 5 goes through a crawl-by transient at the extinction of resource $n=0$. Recovery of resource will begin after a time of approximately

7 $$\begin{aligned} M=O\left( \frac{\log (\varepsilon )}{\log (\delta _p)}\right) . \end{aligned}$$

**Fig. 2:**
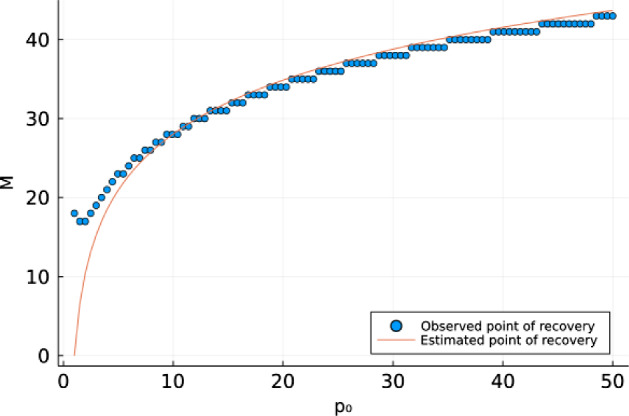
Observed resource recovery time (circles) and estimated recovery time using Eq. 29 (solid line) in System 5 as a function of initial consumer density $p_0$. In this figure, $\delta _p=0.9,\gamma _p=1,\sigma =2.67,\delta _n=0.8,\gamma _n=1,R=2$.

In Fig. 2 we show that Eq. 7 is a reasonably close estimator of the observed point of recovery, especially for large values of the initial consumer density $p_0$, where the consumer density is an order of magnitude higher than the initial resource density $n_0$. We will show in the following theorem that the long-term dynamics shown in Fig. 1 did not depend on the initial conditions of the model.

#### Theorem 2

System 5 has a compact, connected global attractor in the first quadrant $M=\{(p,n)\in \mathbb {R}^2:p\ge 0,n\ge 0\}$.

**Fig. 3:**
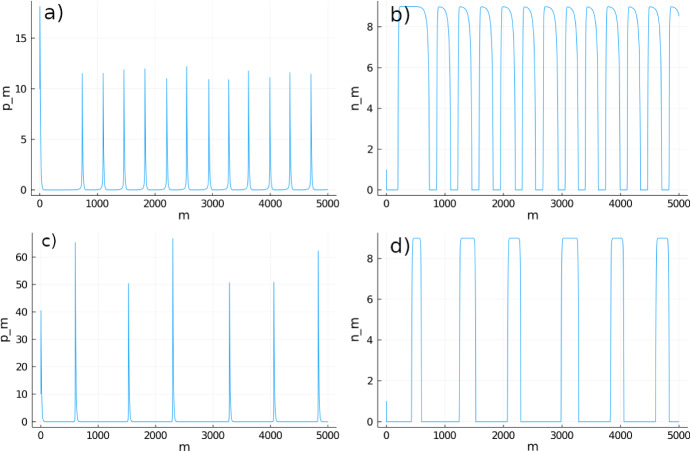
Time series of consumers $p_m$ (**a** and **c**) and resource $n_m$ **b** and **d** following System 5 for 5000 time steps (*m*). In this figure, $p_0=10,n_0=1,\delta _p=0.9,\sigma =2.67,\delta _n=0.8,\gamma _n=1,R=2,$ and the consumer conversion intensity $\gamma _p=3$ (**a** and **b**) and $\gamma _p=8$ (**c** and **d**).

See Appendix C for the proof of this theorem. Theorem 2 implies that, when $\gamma _p<\gamma _p^*$, System 5 will go towards carrying capacity of resource and extinction of consumers. On the other hand, when $\gamma _p>\gamma _p^*$, there are no stable fixed points in the first quadrant. Thus, Theorem 2 implies the existence of a nonlinear attractor, which we can describe based on numerical observations, as shown in Fig. 3.

When $\gamma _p>\gamma _p^*$, the resource population is able to reach a maximum density of carrying capacity and stay there for a prolonged period of time (Fig. 3). However, after the consumer reaches a high enough density, the resource population collapses and passes through a transient extinction phase. This cycle repeats itself through time, but at each repetition, the amplitude of consumer density varies. We hypothetize that this variation in amplitude is caused by the system having a long periodicity. In addition, increasing $\gamma _p$ increases the period between each oscillation. This is consistent with the implication from Theorem 1 that a higher consumer density causes the resource to stay around the extinction equilibrium for a longer period of time.

Figure 3 also shows that the system can stay around the resource-only equilibrium for a prolonged time. We approximate this time in the following theorem, proven in Appendix D.

#### Theorem 3

Let $\gamma _p>\gamma _p^*$, where $\gamma _p^*$ is defined by Eq 6 and let

8 $$\begin{aligned} \lambda _1=\delta _p+\gamma _p\frac{n^*}{1+\sigma n^{*2}}. \end{aligned}$$

Then, if $(p_0,n_0)=(\varepsilon ,n^*-\varepsilon )$ for $0<\varepsilon \ll 1$, System 5 goes a crawl-by transient at the resource-only equilibrium $n=n^*$. resource will start decaying after a time of approximately

9 $$\begin{aligned} M=O\left( \frac{\log \left( \frac{1}{\varepsilon }\right) }{\log (\lambda _1)}\right) . \end{aligned}$$

**Fig. 4:**
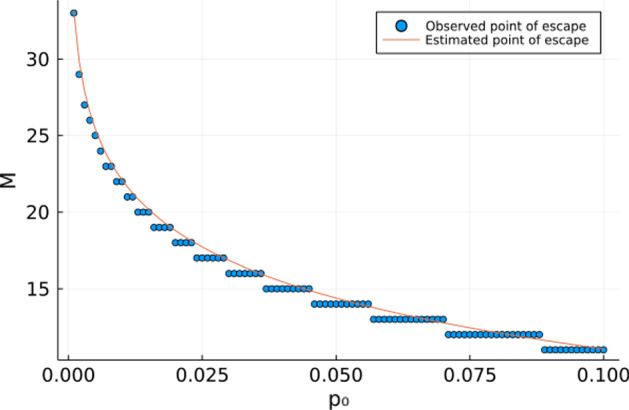
Observed escape time of consumer extinction (circles) and estimated recovery time using Equ 45 (solid line) in System 5 as a function of initial consumer density $p_0$. In this figure, $\delta _p=0.9,\gamma _p=1,\sigma =2.67,\delta _n=0.8,\gamma _n=1,R=2$.

In Fig. 4 we show that this expression is a reasonably close approximation of the time it takes for the consumer to escape extinction across a wide range of orders of magnitude for the initial consumer density.

### Ghost Attractors

**Fig. 5:**
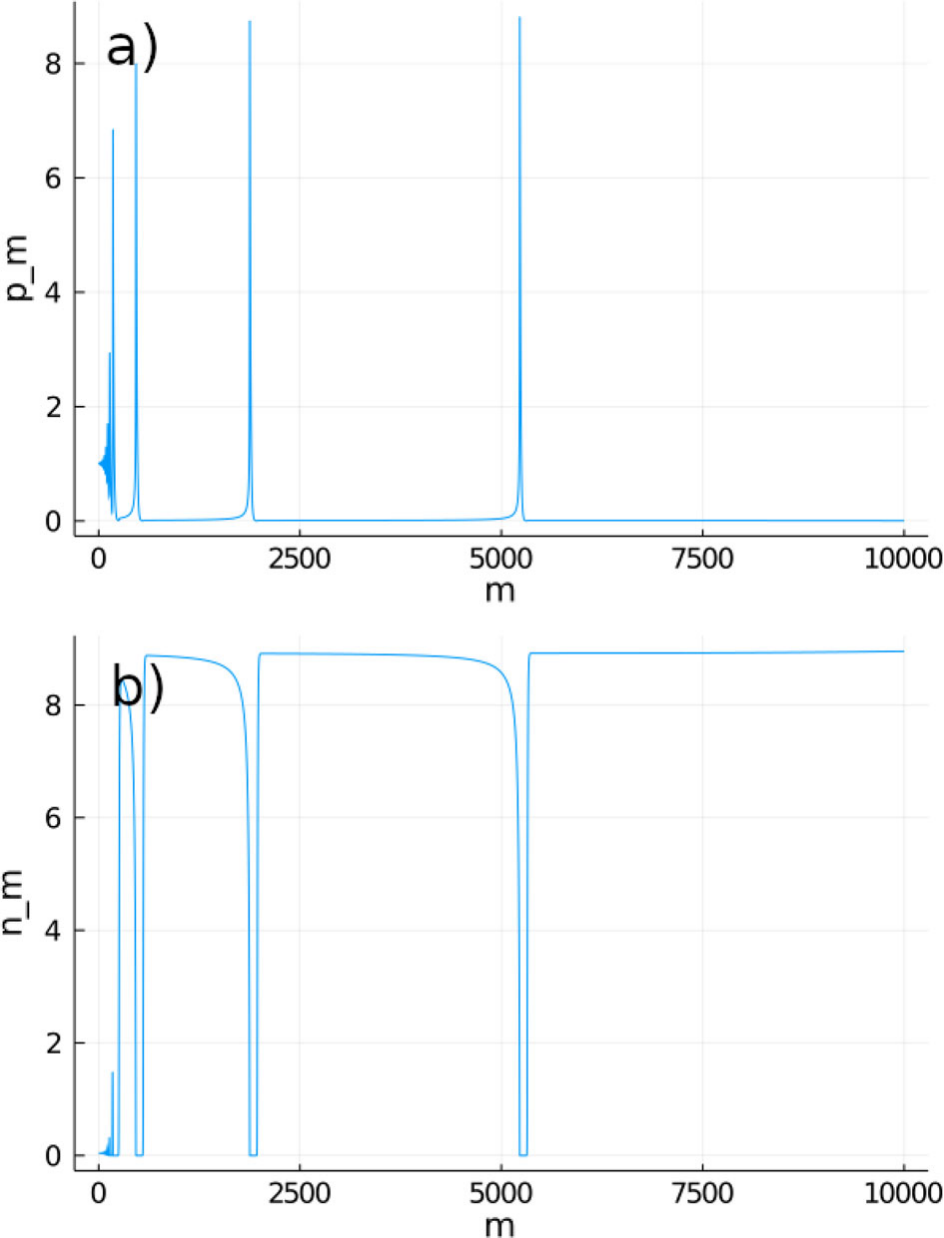
Time series of **a** consumers $p_m$ and **b** resource $n_m$ following System 5 for 10000 time steps. In this figure we consider $p_0=10,n_0=1,\delta _p=0.9,\gamma _p=0.9912\gamma _p^*,\sigma =2.67,\delta _n=0.8,\gamma _n=1,R=2$. Although we know that the system will converge to the equilibrium point, this convergence takes over 5000 time steps.

Theorem 2 ensures that, when $\gamma _p<\gamma _p^*$, System 5 will converge to the stable equilibrium $(0,n^*)$. However, when $\gamma _p^*-\gamma _p\ll 1$, this convergence can take a significantly longer time, as can be seen in Fig. 5. Before the system reaches the equilibrium, the dynamics resemble pseudo-oscillations similar to those observed in Fig. 3 when $\gamma _p>\gamma _p^*$.

Given limitations of available analytical tools for exact derivation of limit cycles in discrete-time models, we approximate the time spent in the ghost attractor $\tau$ by considering a power law for the time spent in a limit cycle (Medeiros et al. 2017):

10 $$\begin{aligned} \tau (\gamma _p)= A(\gamma _p^*-\gamma _p)^{-B}. \end{aligned}$$

**Fig. 6:**
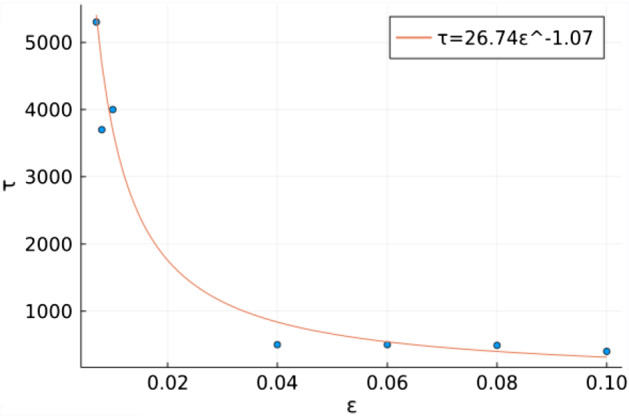
Approximation of transient time ($\tau$) following Eq. 10, where $\varepsilon =\gamma _p^*-\gamma _p$. In this figure, $p_0=10,n_0=1,\delta _p=0.9,\sigma =2.67,\delta _n=0.8,\gamma _n=1,R=2$.

Whenever $n_M>n^\wedge$ and $p_M<p^\wedge$, System 5 shows that $n_{k+1}>n_k$ and $p_{k+1}<p_k$ for all $k>M$. Therefore, we identify the time the system escapes the ghost attractor as $\tau =\min \{M:n_M>n^\wedge ,p_M<p^\wedge \}$. Figure 6 shows that this approximation using the power law provides a reasonable approximation.

## Discussion

In this work we have identified two types of long transients, crawl-by transients and ghost attractors, that can appear in a consumer–resource system with group defense with discrete reproductive pulses. Our long-term dynamics are qualitatively different from those found in Cui et al. (2016), where they identified a variety of bifurcations and chaotic dynamics. The key differences between the two models are that, while that of Cui et al. (2016) models reproduction as a continuous process and integrates the density-dependent growth for a case with overcompensation in discrete time, our model considers reproduction as a discrete event and has a saturating density-dependent function. The model in Cui et al. (2016) is a discrete-time model similar to the Ricker model, where increased reproduction rates lead to unstable dynamics (Ricker 1954). When modeling reproduction as a discrete process with saturating (Beverton–Holt style) density dependence rather than overcompensation, our analysis did not suggest that increased reproduction numbers leads to instabilities in our model.

In addition, discrete reproduction events are one of the main reasons we see the long transients analyzed in this model. The crawl-by transient observed at high consumer densities (Theorem 1) is caused by a sudden crash in the adults of the resource population, which is followed by a slow crash of the consumer population due to its inability to find enough resource for self-replacement. Although the adult resource population is almost nonexistent, the few remaining individuals eventually lead to an increase the resource population when the consumer population becomes small enough.

The other reason long transients appear in this model is due to the self-replacement of consumers depending on the ability of resource to defend themselves. The Type IV Holling functional response produces a bifurcation on the proportionality constant $\gamma _p$ at the value $\gamma _p^*$ given by Eq. 6. This constant can be associated with the conversion capability of consumers, i.e., the amount of energy invested in reproduction activities. When the conversion capability of consumers is too small ($\gamma _p<\gamma _p^*$), group defense of resource will prevent self-replacement of consumers at high resource densities, which will lead to collapse of consumers. When this conversion capability is high enough, self-replacement can be satisfied, and the consumer–resource cycles of Fig. 3 will occur. These cycles and their condition for existence resemble those found in other models where a mechanism of group defense of resource is considered (Ajraldi et al. 2011; Venturino 2011; Venturino and Petrovskii 2013).

In the case where the system presents consumer–resource cycles, the resource-dominated phase will include a crawl-by transient when the conversion capability is close to this bifurcation value (Theorem 3). This will follow by a crash of the resource population, where the consumer-dominated phase appears and presents the crawl-by transient previously described. This behavior presents an alternative perspective to the concept of alternate stable states (Beisner et al. 2003), where the different “alternative stable states” constitute long transients, which may resemble stable states during a long period of time, which then transition into the other phase and stay in a different long transient. In reality, stochasticity may render this juvenile survival when rare impossible or accelerate consumer death, which may lead the model to a stable state in a shorter period of time (Reimer et al. 2021).

When the conversion capability of consumers is smaller than the critical value $\gamma _p^*$ but close to it, these quasiperiodic orbits do not disappear completely and stay as ghost attractors. This ghost attractor stays until the resource density surpasses a given threshold (the equilibrium value $n^\wedge$) and the system enters the basin of attraction of the resource-only equilibrium. The emergence of this ghost attractor is caused by group defense, because in its absence ($\sigma =0$), the unstable equilibria that cause the quasiperiodic orbits $(p^{\vee \wedge },n^{\vee \wedge })$ do not exist. In their absence, resource population density will consistently increase and the consumer density decrease.

The estimation of the transient time of the ghost attractor shows one of the limitations of our analysis, as the theory to study limit cycles in discrete-time systems is not developed enough to precisely analyze the transient time of this ghost attractor. Given the seasonality of the reproduction and recruitment for many organisms (Arreguin-Sanchez 1992; Cameron 1986; Russell et al. 1977; Wallace 1985), a continuous-time model may not properly reflect the biological dynamics we are interested in. Despite this challenge, the expression for the transient time found for transient limit cycles in continuous-time systems in Medeiros et al. (2017) is a reasonably accurate fit in our model. The transient times of the ghost attractor found in our work are similar to those found in the predator–prey model with group defense of Venturino and Petrovskii (2013). However, our biological mechanisms differ, as their transients could be attributed to search time of prey from the predators through space, a feature not explicitly modeled in our work. In contrast, the length of the ghost attractor in our model can be attributed to the length of the crawl-by transients that are part of the cycle itself, which are periods of low population growth for either the consumer or the resource.

In conclusion, we show how long transients can appear in predator–prey systems with group defense and discrete recruitment pulses. A possible extension of this model is to explicitly consider the dynamics of the juvenile stages through a continuous-time model, which could give a more accurate approximation of the transient times found in this paper. A multi-stage model would also allow exploration of the effect of relative adult versus juvenile vulnerability to consumption on the transient dynamics.

## Figures and Tables

**Fig. 7:**
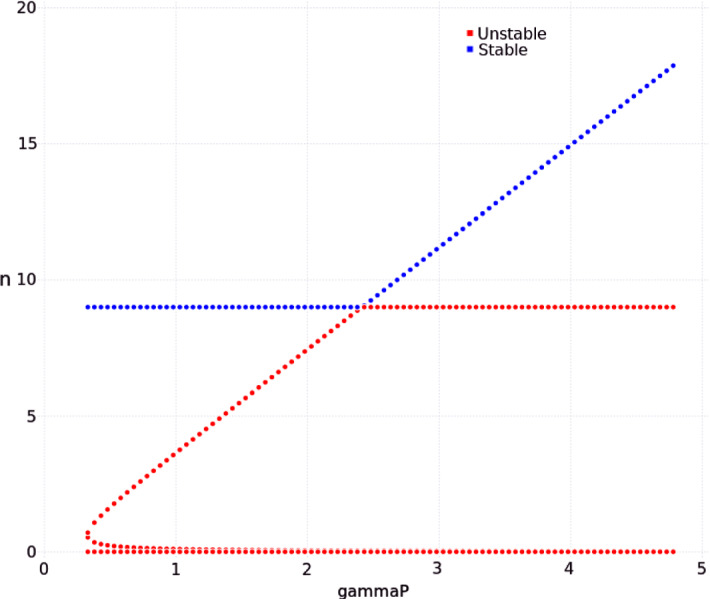
Numerical values of different equilibria for *n* and their stability as we vary $\gamma _p$ in our region of interest. The red points correspond to unstable equilibria, whereas the blue points correspond to stable equilibria. In this figure, $\delta _p=0.9,\sigma =2.67,\delta _n=0.8,\gamma _n=1,R=2$.

## Data Availability

This research does not include any associated data.

## Appendix: A Fixed points of System 5 and their stability

The fixed points of System 5 (*p*, *n*) satisfy the equations

$$\begin{aligned} p=\delta _pp+\gamma _p\frac{pn}{1+\sigma n^2} \end{aligned}$$

$$\begin{aligned} n=\delta _n n\exp \left( -\frac{\gamma _np}{1+\sigma n^2}\right) +Rn\frac{\exp (-p)}{1+n}. \end{aligned}$$

If $p=0$, then the second equation gives us two solutions for *n*, $n=0$ and

11 $$\begin{aligned} n^*=\frac{R}{1-\delta _n}-1.\end{aligned}$$

If $p\ne 0$, then the first equation has two solutions for *n* given by

12 $$\begin{aligned} n^{\vee \wedge }=\frac{\gamma _p}{2(1-\delta _p)\sigma }\left( 1\pm \sqrt{1-\frac{4\sigma (1-\delta _p)^2}{\gamma _p^2}}\right) \end{aligned}$$

where $n^\vee$ corresponds to the solution with a − sign and $n^\wedge$ to the solution with a $+$ sign. These solutions are positive whenever $\gamma _p\ge 2\sqrt{\sigma }(1-\delta _p)$. In such case, plugging $n^{\vee \wedge }$ into the second equation provides us with the following expression:

$$\begin{aligned} \delta _n\exp \left( -\frac{\gamma _n p}{1+\sigma n^{\pm 2}}\right) +\frac{R}{1+n^{\vee \wedge }}\exp (-p)=1. \end{aligned}$$

Then there is an unique value $p^{\vee \wedge }$ that solves the trascendental equation

13 $$\begin{aligned} p^{\vee \wedge }=\log \left( \frac{R}{(1+n^{\vee \wedge })\left( 1-\delta _n\exp \left( -\frac{\gamma _n p^{\vee \wedge }}{1+\sigma n^{\pm 2}}\right) \right) }\right) .\end{aligned}$$

This equation in *p* has an unique solution as the function

14 $$\begin{aligned} f(p)=p-\log \left( \frac{R}{(1+n^{\vee \wedge })\left( 1-\delta _n\exp \left( -\frac{\gamma _n p}{1+\sigma n^{\pm 2}}\right) \right) }\right) \end{aligned}$$

is monotonic for *p* and satisfies $\lim _{p\rightarrow {-\infty }}f(p)<0$ and $\lim _{p\rightarrow {\infty }}f(p)>0$. For it to be biologically relevant, we also require $\lim _{p\rightarrow {0}}f(p)<0$, which will occur when

15 $$\begin{aligned} R>(1-\delta _n)(1+n^{\vee \wedge })\end{aligned}$$

or, after reorganizing the terms, $n^{\vee \wedge }<n^*$.

The Jacobian of the system *J* is the following:

16 $$\begin{aligned} J(p,n)=\left( \begin{array}{cc} \delta _p+\gamma _p\frac{n}{1+\sigma n^2}&{}\gamma _pp\frac{1-\sigma n^2}{(1+\sigma n^2)^2} \\ -\frac{\delta _n\gamma _nn}{1+\sigma n^2} \exp \left( -\frac{\gamma _np}{1+\sigma n^2}\right) -\frac{Rn\exp (-p)}{1+n}&{} \delta _n\exp \left( -\frac{\gamma _np}{1+\sigma n^2}\right) \left( 1+\frac{2\sigma \gamma _n pn^2}{(1+\sigma n^2)^2}\right) +\frac{R\exp (-p)}{(1+n)^2} \end{array}\right) .\end{aligned}$$

From here, the extinction equilibrium satisfies

17 $$\begin{aligned} J(0,0)=\left( \begin{array}{cc} \delta _p &{} 0\\ 0 &{} \delta _n+R \end{array}\right) \end{aligned}$$

which has eigenvalues $\delta _p<1$ and $\delta _n+R>1$. Therefore the extinction equilibrium is a saddle. For the resource-only equilibrium, the upper right term of the Jacobian matrix equals 0 whenever $p=0$. Therefore $J(0,n^*)$ is a triangular matrix, with the eigenvalues being the diagonal terms

18 $$\begin{aligned} \lambda _1=\delta _p+\gamma _p\frac{n^*}{1+\sigma n^{*2}},\end{aligned}$$

19 $$\begin{aligned} \lambda _2=\delta _n+\frac{(1-\delta _n)^2}{R}.\end{aligned}$$

Because $R>1-\delta _n$, $0<\lambda _2<1$. $\lambda _1$, on the other hand, $\lambda _1$ will produce a change in stability when

20 $$\begin{aligned} \gamma _p=\gamma _p^*=(1-\delta _p)\frac{1+\sigma n^{*2}}{n^{*}}.\end{aligned}$$

In this case, the equilibrium is stable whenever $\gamma _p<\gamma _p^*$ and a saddle when $\gamma _p>\gamma _p^*$. Because $n^*>1/\sqrt{2\sigma }$, plugging $\gamma _p=\gamma _p^*$ in Eq. 12, we have that $n^*=n^\wedge$. Based on the conditions for $(p^\wedge ,n^\wedge )$ to be biologically reasonable, this implies that at $\gamma _p=\gamma _p^*$, the system goes through a transcritical bifurcation, where the carrying capacity $(0,n^*)$ changes stability.

The trascendental equation that describes $p^{\vee \wedge }$ renders it impossible to analyze them directly. However, a numerical exploration in Fig. A.6 shows that these equilibria are unstable for $\gamma _p<\gamma _p^*$ and $(p^\wedge ,n^\wedge )$ becomes stable for $\gamma _p>\gamma _p^*$. Combining this result with the condition for the equilibrium point $(p^\wedge ,n^\wedge )$ to be biologically relevant (Eq. 15), we find that when $\gamma _p>\gamma _p^*$, there are no stable fixed points in the first quadrant (i.e., $\mathbb {R}^2_+$).

This transcritical bifurcation occurs with almost any combination of parameters in our region of interest. To show this, we perform a similar analysis as those in Khan et al. (2016); Murakami (2007). When $\gamma _p=\gamma _p^*$, we can rewrite our system in diagonal form and centered around the origin by making the change of variables:

21 $$\begin{aligned} x_m=\frac{\lambda _2-1}{n^*\left( 1-\delta _n+\frac{\delta _n\gamma _n}{1+\sigma n^{*2}}\right) }p_m\end{aligned}$$

22 $$\begin{aligned} y_m=p_m+n_m-n^*\end{aligned}$$

provided that $\left( 1-\delta _n+\frac{\delta _n\gamma _n}{1+\sigma n^{*2}}\right) \ne 0$. Otherwise, we let $x_m=p_m,y_m=n_m-n^*$. In both cases, this lets use write our System 5 as

23 $$\begin{aligned} \left( \begin{array}{c} x_{m+1} \\ y_{m+1} \end{array}\right) =\left( \begin{array}{cc} 1 &{} 0 \\ 0 &{} \lambda _2 \end{array}\right) \left( \begin{array}{c} x_{m} \\ y_{m} \end{array}\right) +\hbox {h.o.t}.\end{aligned}$$

We can expand this system to include the parameter as a dynamical factor with eigenvalue 1 as $\mu _m\equiv \gamma _p-\gamma _p^*$. The central limit theorem gives us that $y_m=h(x_m,\mu _m)$ for some function $h=O((x_m+\mu _m)^2)$. Because $x_m$ is a multiple of $p_m$, its dynamics follow the same trend and can be approximated up to $O((x_m+\mu _m)^3)$ as:

24 $$\begin{aligned} x_{m+1}=f(x_m,\mu _m)=x_m+\frac{\gamma _p^*(1-\sigma n^{*2})}{(1+\sigma n^{*2})^2}x_m^2+\frac{\sigma n^{*2}(n^*-\gamma _p^*)}{(1+n^{*2})^2}x\mu _m+O((x_m+\mu _m)^3)\end{aligned}$$

Equation A.14 satisfies that $f_x(0,0)=1,f_\mu (0,0)=0,f_{xx}(0,0)\ne 0$ Because we assume that $n^*>1/\sqrt{\sigma }$, and $f_{x\mu }(0,0)\ne 0$ except when $n^*=\gamma _p^*$. Plugging this value Eq. 6, we get that the condition $n^*=\gamma _p^*$ holds only when $n^*= \sqrt{\frac{1-\delta _p}{\sigma \delta _p}}$.

Therefore, whenever $n^*\ne \sqrt{\frac{1-\delta _p}{\sigma \delta _p}}$, the system goes through a transcritical bifurcation between $(0,n^*)$ and $(p^\wedge ,n^\wedge )$ as $\gamma _p$ passes through $\gamma _p^*.$

## Appendix: B Proof of Theorem 1

If $p_0=O(\varepsilon ^{-1})$, plugging $O(\varepsilon ^{-1})$ into the formula for $n_1$ gives us that $n_1=o(\varepsilon )$. Plugging $o(\varepsilon )$ into the formula for $p_2$ gives us that:

25 $$\begin{aligned} p_2=\delta _pp_1+o(\varepsilon ).\end{aligned}$$

In addition, if $n_m=o(\varepsilon )$, the equation for $n_{m+1}$ satisfies:

26 $$\begin{aligned} \frac{n_{m+1}}{n_m}=O\left( \delta _n\exp \left( -\gamma _n p_m\right) +R\exp (-p_m)\right) .\end{aligned}$$

While $p_m=O(\varepsilon ^{-1})$, this expression will satisfy $n_{m+1}/n_m<1$. We can thus assume that the expression

27 $$\begin{aligned} p_{m+1}=\delta _p p_m+o(\varepsilon )\end{aligned}$$

is satisfied until $p_{m+1}=O(1)$. Therefore, when $p_m=O(\varepsilon ^{-1})$, the consumer population time evolution can be approximately solved as

28 $$\begin{aligned} p_m=\frac{\delta _p^m}{\varepsilon }+o(\varepsilon ).\end{aligned}$$

This expression stops working when $p_m=O(1)$, and thus resource will start a recovery afterwards. We can estimate the order of magnitude of such *m* by plugging $p_m=1$ above. Solving for *m* gives us that

29 $$\begin{aligned} m=\frac{\log (\varepsilon )}{\log (\delta _p)}\end{aligned}$$

which is the expression that proves the theorem.

## Appendix: C Proof of Theorem 2

To show the existence of this theorem, we use Theorem 2.9 of Magal and Zhao (2005). To do this, we consider the first quadrant *M* as a metric subspace of $\mathbb {R}^2$ with metric $d(x,y)=\Vert x-y\Vert _2$ induced by the Euclidean norm. Let $T:M\rightarrow M$ be given by

30 $$\begin{aligned} T\left( \begin{array}{c} p\\ n \end{array}\right) =\left( \begin{array}{c} p\left( \delta _p+\frac{\gamma _p n}{1+\sigma n^2}\right) \\ n\left( \delta _n\exp \left( -\frac{\gamma _np}{1+\sigma n^2}\right) +\frac{R\exp (-p)}{1+n}\right) \end{array}\right) .\end{aligned}$$

We show that *T* is a point dissipative, compact map on *M*. Because *M* is a subspace of $\mathbb {R}^2$, compactedness is trivial. A map is point dissipative if there is a bounded set $B_0\subset M$ such that $B_0$ attracts each point in *M*. To show *T* is point dissipative, we find such bounded set $B_0$.

Let

31 $$\begin{aligned} \begin{aligned}f_p(n)&=\delta _p+\frac{\gamma _p n}{1+\sigma n^2},\\f_n(p,n)&=\delta _n\exp \left( -\frac{\gamma _np}{1+\sigma n^2}\right) +\frac{R\exp (-p)}{1+n}.\end{aligned}\end{aligned}$$

Note that $f_n<1$ whenever $n>n^*$, where $n^*$ is given by Equation 11. This implies that *n* is attracted by the set $[0,n^*]$. Without loss of generality, we assume that $n\in [0,n^*]$. Suppose that $p>p^*$, where $p^*$ is

32 $$\begin{aligned} p^*=\max \left( \frac{1+\sigma n^{*2}}{\gamma _n},1\right) =\frac{1}{\varepsilon }. \end{aligned}$$

A similar argument to that of the proof for Theorem 1 shows that in this case, the consumer population will satisfy $p=O(1)$ in time $\ln (\varepsilon )/ln(\delta _p)$. In particular, $p<p^*$ after a period of time. In addition, if $p<p^*$ but $Tp>p^*$, $f_p$ satisfies:

33 $$\begin{aligned} f_p(n)\le \delta _p+\frac{\gamma _p}{2\sqrt{\sigma }}\end{aligned}$$

for any *n*. This implies that $Tp\le \left( \delta _p+\frac{\gamma _p}{2\sqrt{\sigma }}\right) p^*$. Let $\tau$ be the period of time such that $T^\tau \left( \delta _p+\frac{\gamma _p}{2\sqrt{\sigma }}\right) p^*<p^*$. Therefore *p* is attracted by the set $[0,(\delta _p+\frac{\gamma _p}{2\sqrt{\sigma }})^\tau p^*]$. Then, the bounded rectangle

34 $$\begin{aligned} B_0:=\left[ 0,\left( \delta _p+\frac{\gamma _p}{2\sqrt{\sigma }}\right) ^\tau p^*\right] \times [0,n^*] \end{aligned}$$

is an attracting set in *M*. Therefore, *T* is a point dissipative map.

Therefore, Theorem 2.9 of Magal and Zhao (2005) implies that there is a compact global attractor in *M*. Finally, because *M* is locally connected, Theorem 4.5 of Gobbino and Sardella (1997) implies that the global attractor is connected, which completes the proof.

## Appendix: D Proof of Theorem 3

Let $x_m=p_m,y_m=n^*-n_m$. Because $\Vert (x_m,y_m)\Vert =O(\varepsilon )$, System 5 can be approximated by the linearized system:

35 $$\begin{aligned} \left( \begin{array}{c} x_{m+1} \\ y_{m+1} \end{array}\right) \sim J(0,n^*)\left( \begin{array}{c} x_{m} \\ y_{m} \end{array}\right) ,\end{aligned}$$

where the Jacobian $J(0,n^*)$ is described by Eq. 16. The calculations of Appendix A show that the Jacobian $J(0,n^*)$ is a lower triangular matrix, with eigenvalues

36 $$\begin{aligned} \lambda _1=\delta _p+\gamma \frac{n^*}{1+\sigma n^{*2}}.\end{aligned}$$

37 $$\begin{aligned} \lambda _2=\delta _n+\frac{(1-\delta _n)^2}{R}\end{aligned}$$

and eigenvectors

38 $$\begin{aligned} v_1=\left( \begin{array}{c} u \\ 1 \end{array}\right) ,v_2=\left( \begin{array}{c} 0 \\ 1 \end{array}\right) \end{aligned}$$

where

39 $$\begin{aligned} u=\frac{\lambda _2-\lambda _1}{n^*\left( 1-\delta _n+\frac{\delta _n\gamma _n}{1+\sigma n^{*2}}\right) }. \end{aligned}$$

This system has for solution the expression

40 $$\begin{aligned} \left( \begin{array}{c} x_m \\ y_m \end{array}\right) =a\lambda _1^mv_1+b\lambda _2^mv_2,\end{aligned}$$

where *a*, *b* are constants. If we let $m=0$, then we can solve the linear system

41 $$\begin{aligned} \left( \begin{array}{c} \varepsilon \\ \varepsilon \end{array}\right) =\left( \begin{array}{cc} u&{}0 \\ 1&{}1 \end{array}\right) \left( \begin{array}{c} a \\ b \end{array}\right) ,\end{aligned}$$

which has solutions

42 $$\begin{aligned} \left( \begin{array}{c} a \\ b \end{array}\right) =\left( \begin{array}{cc} \frac{1}{u}&{}0 \\ -\frac{1}{u}&{}1 \end{array}\right) \left( \begin{array}{c} \varepsilon \\ \varepsilon \end{array}\right) .\end{aligned}$$

In particular, this gives us that $a=\frac{\varepsilon }{u}$. Because $\gamma _p>\gamma _p^*$, then $\lambda _1>1$, and $\lambda _2<1$. Therefore, for big *m*, System 40 can be approximated as

43 $$\begin{aligned} \left( \begin{array}{c} x_m \\ y_m \end{array}\right) \sim a\lambda _1^mv_1=\frac{\varepsilon \lambda _1^m}{u}\left( \begin{array}{c} u \\ 1 \end{array}\right) .\end{aligned}$$

Thus, System 5 will stay near the resource-only equilibrium as long as $\Vert (x_m,y_m)\Vert =O(\varepsilon )$. In particular, the System will escape the saddle point when $x_m=O(1)$. Plugging in $x_m=1$ into the approximated solution lets us find *M* that solves the equation

44 $$\begin{aligned} 1=\varepsilon \lambda _1^M.\end{aligned}$$

This has for solution

45 $$\begin{aligned} M=\frac{\log \left( \frac{1}{\varepsilon }\right) }{\log (\lambda _1)}\end{aligned}$$

which is the expression that proves the theorem.
